# Supplementary material for: Irminger Sea deep convection injects oxygen and anthropogenic carbon to the ocean interior
Source: Nat Commun. 2016 Oct 27;7:13244. doi: 10.1038/ncomms13244 (PMC5095284; doi:10.1038/ncomms13244)
Supplement: Supplementary Information — Supplementary Figures 1-5 [file ncomms13244-s1.pdf]

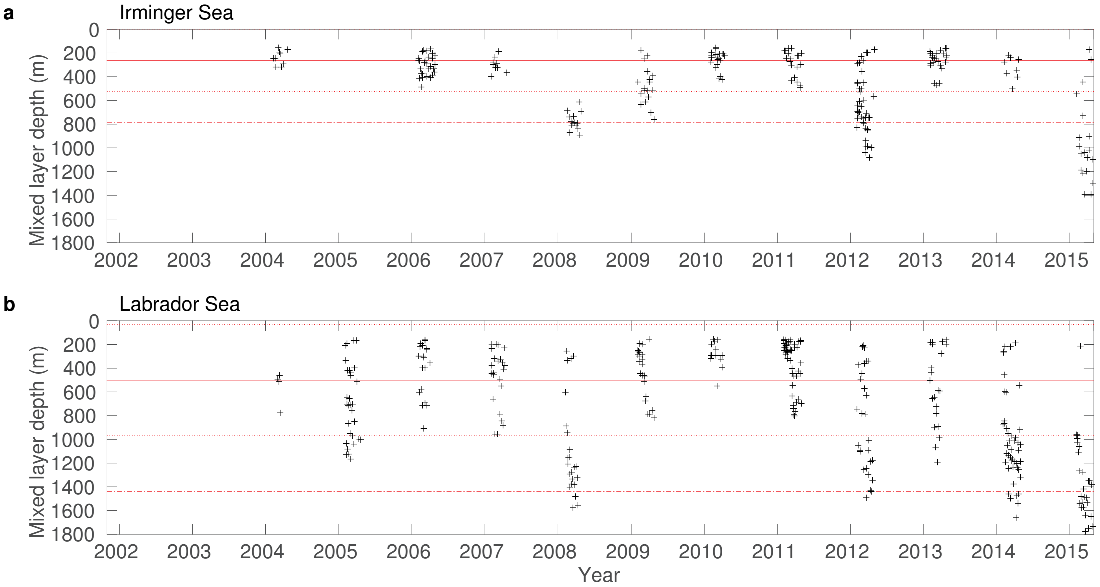

Supplementary Fig. 1: **Mixed layer depth Irminger Sea - Labrador Sea.** February-April mixed layer depth estimates within 80% of deepest recorded mixed layer depth for individual Argo floats for **a** the Irminger Sea and **b** the Labrador Sea (55.6-59 °N, 55-46°W) over 2002 to 2015. Mixed layer depths more shallow than 150 m were excluded. The solid red lines represent the mean mixed layer depth, red dotted lines ± one standard deviation and dashed-dotted lines two standard deviations. The largest deviation from the long-term mean is observed in the Irminger Sea in winter 2014-15.

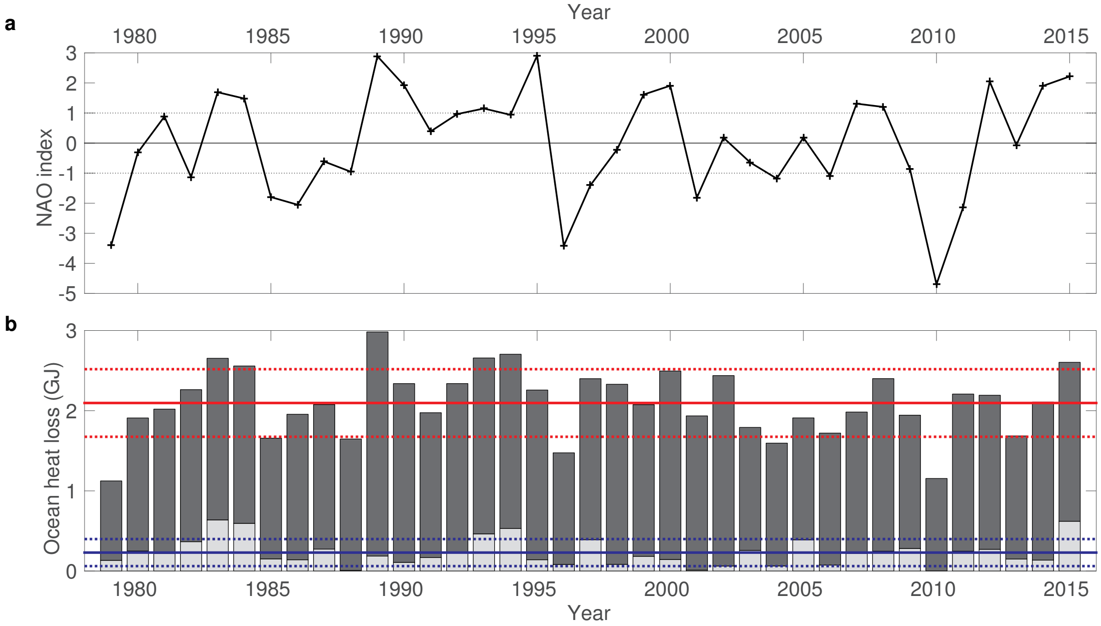

Supplementary Fig. 2: **NAO index and oceanic heat loss over the Irminger Sea. a,** December to February NAO index from 1979 to 2015 based on ERA-Interim reanalysis data. **b,** The winter mean ocean heat loss over the Irminger Sea from November to April from 1979 to 2015 based on ERA-Interim reanalysis data. The light grey shading indicates the heat loss associated with tip jet events. The red solid line is the mean ocean heat loss over 1979 to 2015, the red dotted lines represent ± one standard deviation. The blue solid line is the mean ocean heat loss associated with tip jet events over 1979 to 2015, the blue dotted lines represent ± one standard deviation.

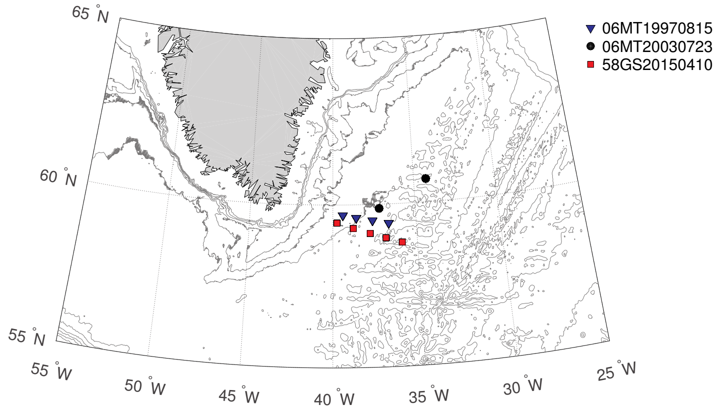

Supplementary Fig. 3: **Location of stations of cruise data.** Selected stations for Fig.4 for 06MT19970815, 06MT20030723 and 58GS20150410 cruise data in the Irminger Sea. Depth contours every 500 m are shown.

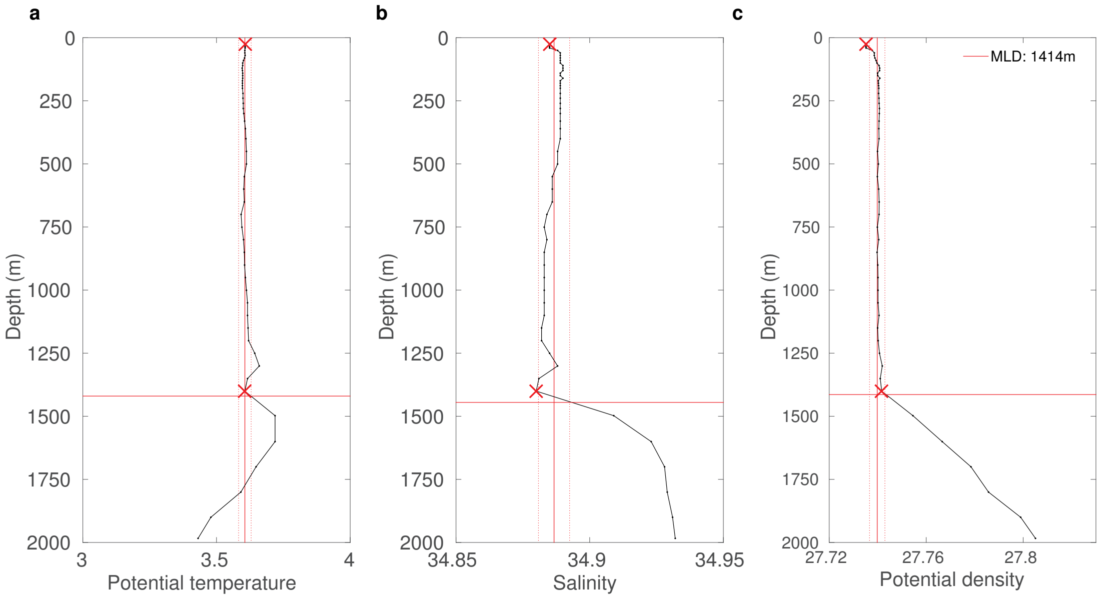

Supplementary Fig. 4: **Mixed layer depth estimates.** Mixed layer depth (MLD) determination for an example Argo profile of **a** potential temperature, **b** salinity and **c** potential density from April 16th, 2015 in the Irminger Sea region. The dotted red lines are the two-standard deviation envelope of the mean over the depth range indicated by the red crosses, close to the mixed layer depth. The intercept of original profile with two-standard deviation envelope marks the mixed layer depth.

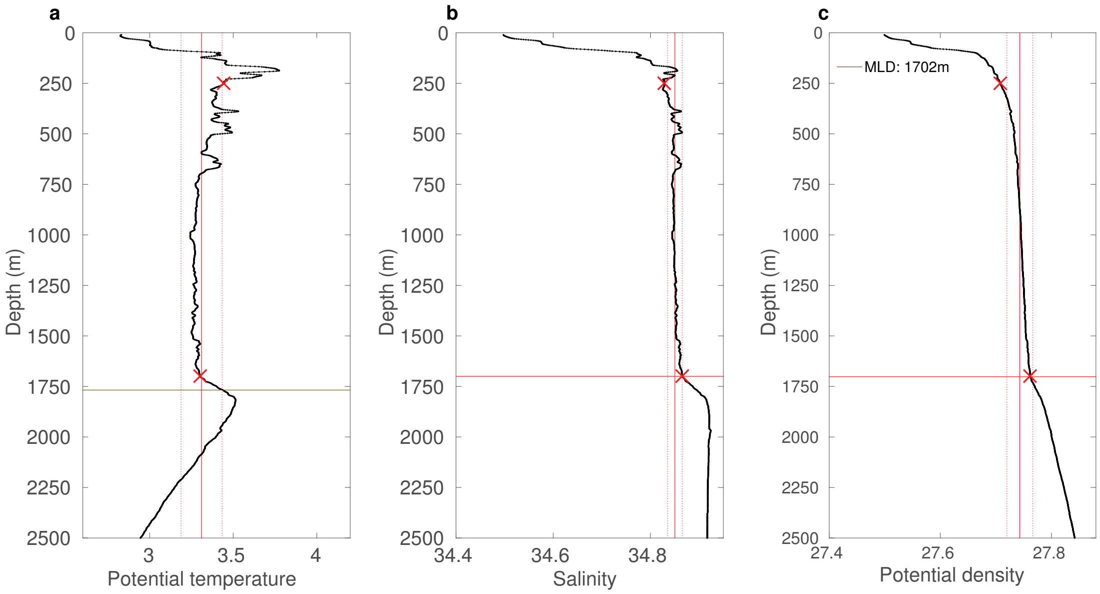

Supplementary Fig. 5: **Estimate of mixed layer depth isolated from the surface.** Mixed layer depth (MLD) determination for a mixed layer that is isolated from the surface for an example CTD profile of **a** potential temperature, **b** salinity and **c** potential density from May 13th, 2015 in the Labrador Sea. The dotted red lines are the two-standard deviation envelope of the mean over the depth range indicated by the red crosses, close to the mixed layer depth. The intercept of original profile with two-standard deviation envelope marks the mixed layer depth.
